# Preliminary characterization of gut mycobiome enterotypes reveals the correlation trends between host metabolic parameter and diet: a case study in the Thai Cohort

**DOI:** 10.1038/s41598-024-56585-2

**Published:** 2024-03-09

**Authors:** Kevin Mok, Thitirat Poolsawat, Surasawadee Somnuk, Bandhita Wanikorn, Preecha Patumcharoenpol, Sunee Nitisinprasert, Wanwipa Vongsangnak, Massalin Nakphaichit

**Affiliations:** 1 Department of Biotechnology, Faculty of Agro-Industry, Kasetsart University, Bangkok, 10900 Thailand; 2 Specialized Research Unit: Probiotics and Prebiotics for Health, Faculty of Agro-Industry, Kasetsart University, Bangkok, 10900 Thailand; 3 Specialized Research Unit: Functional Food and Human Health Laboratory, Faculty of Agro-Industry, Kasetsart University, Bangkok, 10900 Thailand; 4 Department of Sports and Health Sciences, Faculty of Sport Science, Kasetsart University, Kamphaeng Saen Campus, Nakhon Pathom, 73140 Thailand; 5 Department of Zoology, Faculty of Science, Kasetsart University, Bangkok, 10900 Thailand; 6 Omics Center for Agriculture, Bioresources, Food, and Health, Kasetsart University (OmiKU), Bangkok, 10900 Thailand

**Keywords:** Microbiome, Fungi, Metagenomics

## Abstract

The association between the gut mycobiome and its potential influence on host metabolism in the Thai Cohort was assessed. Two distinct predominant enterotypes, *Saccharomyces* (Sa) and *Aspergillus*/*Penicillium* (Ap/Pe) showed differences in gut mycobiota diversity and composition. Notably, the Sa enterotype exhibited lower evenness and richness, likely due to the prevalence of *Saccharomyces*, while both enterotypes displayed unique metabolic behaviors related to nutrient metabolism and body composition. Fiber consumption was positively correlated with adverse body composition and fasting glucose levels in individuals with the Sa enterotype, whereas in the Ap/Pe enterotype it was positively correlated with fat and protein intake. The metabolic functional analysis revealed the Sa enterotype associated with carbohydrate metabolism, while the Ap/Pe enterotype involved in lipid metabolism. Very interestingly, the genes involved in the pentose and glucuronate interconversion pathway, such as polygalacturonase and l-arabinose-isomerase, were enriched in the Sa enterotype signifying a metabolic capacity for complex carbohydrate degradation and utilization of less common sugars as energy sources. These findings highlight the interplay between gut mycobiome composition, dietary habits, and metabolic outcomes within the Thai cohort studies.

## Introduction

Numerous studies have established a connection between gut microbiota and host well-being^1–5^. The gut microbiota, comprising bacteria, fungi, viruses, and archaea form a complex ecosystem that coexists with the host within the gastrointestinal tract. The role of gut bacteria has been extensively explored in terms of nutrient metabolism, immune modulation, and pathogen defense^6–8^. However, the significance of gut fungi, also known as the gut mycobiome, has received comparatively less attention.

Recent studies highlighted the predominance of *Ascomycota* and *Basidiomycota* phyla, constituting 70–95% of the human gut mycobiome across diverse geographical contexts^9–12^. Genera such as *Aspergillus*, *Penicillium*, *Saccharomyces*, *Malassezia*, and *Candida* have been identified within the gastrointestinal mycobiome. Mishima et al. revealed an age-associated transition from environmentally-sourced fungal taxa to yeast-like species in infants^13^. This result concurred with a previous in vivo study^14^, where advancing age was correlated with a reduction of gut mycobiome diversity, thus accentuating the interplay between host-related factors and fungal community composition.

Recent reports have also highlighted the significant contribution of gut fungi in maintaining gut integrity and stability^15,16^. Gut fungi and gut bacteria actively participate in nutrient digestion and metabolism, particularly in the breakdown of complex carbohydrates and fibers that are otherwise indigestible by human enzymes^17^. Certain gut fungi also play a critical role in immune system development and function^6,18^. Fungal cell-wall components, proteins and their secreted metabolites interact with the host immune system, influencing the maturation and activation of immune cells^19,20^. Perturbations of gut mycobiota have been associated with various diseases including inflammatory bowel disease (IBD), obesity, allergies, and even neurological disorders^18,21–23^ but comprehensive studies on how gut fungi influence host well-being are lacking and the extent of their contribution to overall host health remains unclear.

A previous study explored the concept of “enterotypes” as the major microbiota populations in the human gut that influence the overall well-being of the host^24,25^. The study participants consumed identical diets but variations in the predominant colonizers within their gut microbiota led to divergent physiological behaviors. For instance, individuals harboring a *Prevotella* enterotype exhibited heightened capabilities in digesting a high-fiber diet^26^, while those with a *Bacteroides* enterotype, characterized by elevated saccharolytic and proteolytic digestive enzymes^27^, had differing energy uptake profiles. Studies involving gut microbiota transplantation in mice found that recipient mice fed identical diets before transplantation exhibited obesity induction following the introduction of distinct gut microbiota^28^.

The precise impact of diet on gut mycobiota composition remains unclear, with several studies undertaken to elucidate the nature of this relationship. One such study involving mice revealed that the consumption of ultra-processed diets characterized but high carbohydrates but low fiber was linked to a reduction in the diversity of the gut mycobiome^14^. A decrease in *Saccharomyces* abundance was also reported as a result of ultra-processed diet consumption, suggesting that the interplay between the host and microbial metabolic processes might be influenced by the composition of the gut mycobiome.

Here, we hypothesized that a similar relationship might exist between the gut mycobiome and host metabolism. Alongside gut bacteria, gut fungi also contribute to enhanced energy uptake and promote weight gain in individuals with specific gut mycobiota profiles^6,14,29^. Gut mycobiota play a role in energy metabolism, with a potential impact on weight regulation but the precise mechanisms underlying these relationships require further elucidation. Hence, to better understand the intricate interplay between the gut mycobiota and host energy homeostasis, this study comprehensively explored the distinctive variations in gut mycobiome composition and functional activities and examined their potential implications for host metabolism and digestion.

## Results

### Gut mycobiome diversity in Thai cohorts

ITS2 metagenomics sequencing was performed to determine the diversity of gut mycobiota. The sequencing process yielded an average of 127,774 sequences per sample, ranging from 8407 to a maximum of 634,034 reads across all samples. To facilitate diversity analysis, the sequence was normalized based on the rarefaction curve to 8407 sequences per sample. For taxonomy analysis the relative abundance was determined by dividing reads from each amplicon sequence variant (ASV) by the total reads within each respective sample. The unidentified taxa at the phylum level were classified as fungi species which on average compromise 12.85% of the overall abundance. Overall, 1083 ASVs were recorded in all samples, with representation from 4 phyla, 23 classes, and 126 genera. Data of mycobiota compromise at least 0.01% of total abundance can be found in Tables S1, S2, and S3.

Three phyla were present across all samples, with abundance from highest to lowest as *Ascomycota*, *Basidiomycota*, and *Mucoromoycota*. At the class level, the most prominent commensal fungi were identified as *Saccharomycetes*, *Eurotiomycetes*, and *Dothideomycetes*. Interestingly, the distribution abundance of *Saccharomycetes* and *Eurotiomycetes* covered a wide range spanning 0.73–98.07% and 0.62–80.75%, respectively. Observation revealed *Aspergillus* and *Penicillium* as the predominant genera within the class *Eurotiomycetes*, while *Saccharomyces* emerged as the prominent genus within the class *Saccharomycetes*.

### Gut mycobiome enterotype in the Thai cohorts

A cluster analysis was performed to better understand the large differences in gut mycobiome enterotype abundance within the Thai cohort. The clustering process involved the utilization of the Bray–Curtis dissimilarity hierarchical cluster analysis and an unsupervised clustering method employing the partitioning around medoids (PAM) algorithm^24^. The optimal number of clusters for the unsupervised approach was determined to be two, as indicated by the analysis of the Calinski-Harabasz (CH) index (Fig. S1).

**Figure 1:**
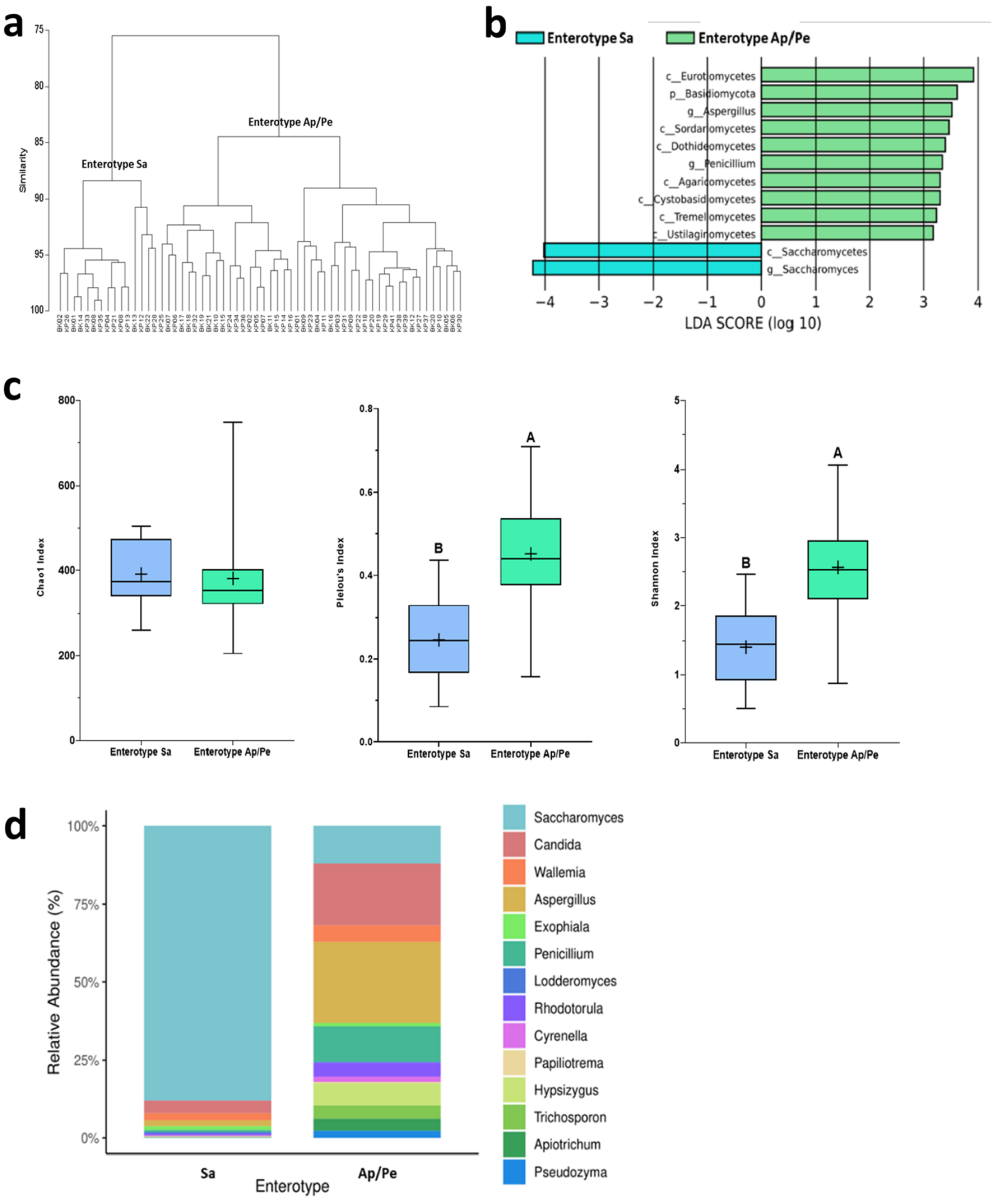
Distinct clustering patterns of gut mycobiota in Thai adults based on Bray–Curtis dissimilarity hierarchical cluster analysis (**a**), revealing specific taxa contributing significantly to each cluster in LEFSe analysis (**b**). Gut mycobiome alpha diversity (**c**) and profile (**d**) in Thai adults with Saccharomyces (Sa) and Aspergillus/Penicillium (Ap/Pe) enterotypes were visualized. Alpha diversity was presented in box and whisker plots, indicating statistically significant differences (p < 0.05) denoted by different letters (A, B). The bar graph depicts the genera-level taxa with an average relative abundance higher than 0.1%.

Among the study participants, two distinct clusters emerged, each driven by the abundance of gut mycobiota and referred to as enterotypes. The linear discriminant analysis effect size (LEfSe) further identified these enterotypes, revealing that the first cluster, referred to as the Sa enterotype, was predominantly influenced by the *Saccharomyces* genus. In contrast, the second cluster, designated as the Ap/Pe enterotype, exhibited a higher association with *Aspergillus*/*Penicillium*, as identified by specific mycobionts (Fig. 1a,b). The outcomes of the analysis showcased that 15 samples were categorized into cluster 1 (Sa enterotype), while the remaining 45 samples formed the Ap/Pe enterotype (Fig. S2).

Alpha diversity analysis showed that individuals classified under the Sa enterotype exhibited notably lower gut mycobiont evenness and diversity compared to those within the Ap/Pe enterotype (Fig. 1c). Interestingly, no significant disparity was observed in the richness index. Although infrequently reported, high gut mycobiota diversity has been linked to the manifestation of various diseases including atopic allergies, inflammatory bowel disease, and colorectal cancer^5,30,31^.

Further investigation, focusing on the genus level (Fig. 1d), showed that individuals within the Sa enterotype displayed a high abundance of *Saccharomyces* species, ranging from 60 to 93%. By contrast, the Ap/Pe enterotype demonstrated more even diversity, predominantly comprising six genera: *Aspergillus, Candida, Penicillium, Saccharomyces, Wallemia,* and *Rhodotorula*. Among these, *Saccharomyces*, *Aspergillus*, and *Penicillium* have been frequently reported as commensal fungi typically found in the human gut^9,32,33^, with *Candida*, *Wallemia*, and *Rhodotorula* associated with disease exacerbation in individuals with compromised immune systems^18,31,34^.

The clustering of enterotypes strongly corresponded to the abundance of *Saccharomyces* and *Aspergillus*, and differences at the species level were examined within each enterotype. Three predominant species with prevalence exceeding 40% were identified in each enterotype, namely *Aspergillus gracilis*, *Aspergillus restrictus*, and *Aspergillus clavatus*. A higher prevalence of *Aspergillus caesiellus* among individuals exhibiting the Sa enterotype (29% prevalence compared to 15% in the Ap/Pe enterotype) was also observed. However, the ITS2 sequences were unable to distinguish between species within the *Saccharomyces* genus.

### Dietary habits and lifestyle of the study participants

Commensal gut mycobiota are generally regarded as having a neutral impact on the host but they have also been shown to actively participate in host energy metabolism^6^. Within the bacterial counterpart, the enterotype impacts host well-being and metabolic activity in different ways^24^. Therefore, we investigated the potential influence of enterotypes on host metabolic processes, taking note of the variations in nutrition intake between each group. Results showed no significant differences in energy, water, macronutrient, and micronutrient consumption between individuals within each enterotype (Table 1). In addition, the preferred foods of individuals in both enterotypes showed no significant differences (p > 0.05) based on food types, including vegetables, fruits, dairy products, soybean products, processed meats, offal, seafood, eggs, fish, red meat, chicken, cakes and sweets, snacks (chips and crackers), butter, and coconut (Fig. S3). The results of the lifestyle analysis were also evaluated, revealing no significant differences (p > 0.05) in the level of activity between the two enterotype groups (Fig. S4).

**Table 1:** Nutrition parameter differences between each enterotype.

Nutrition parameter	Enterotype		p-value
	Sa (n = 15)	Ap (n = 45)	
Energy	1305.74 ± 373.1	1414.56 ± 563.33	0.406
Total carbohydrate	169.98 ± 63.07	158.14 ± 58.18	0.533
Sugar	162.6 ± 112.13	124.56 ± 77.53	0.237
Total fat	44 ± 16.93	56.04 ± 30.05	0.067
Cholesterol	847 ± 406.47	980.36 ± 576.92	0.338
Saturated fat	40.85 ± 16.26	44.88 ± 29.42	0.516
Total protein	57.46 ± 21.1	69.41 ± 36.79	0.131
Protein from animal	39.28 ± 19.13	50.55 ± 33.85	0.119
Protein from vegetable	10.39 ± 4.68	10.25 ± 5.41	0.927
Crude fiber	0.99 ± 2.04	0.44 ± 0.58	0.314
Dietary fiber	21.81 ± 14.46	20.72 ± 10.73	0.793
Water	2401.9 ± 1553.53	2828.88 ± 1790.54	0.389

**Figure 2:**
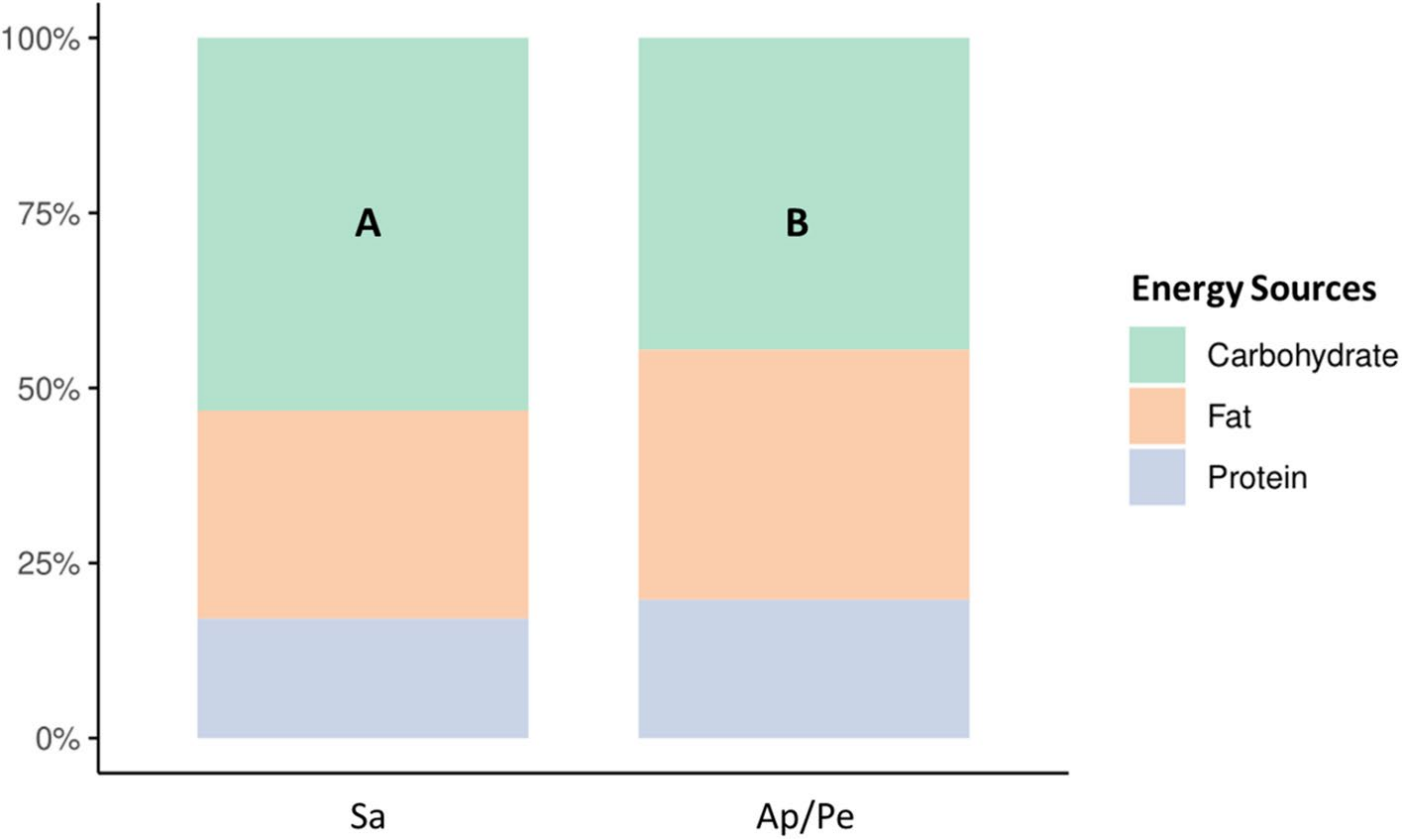
Bar chart showing differences in energy proportion derived from the intake of three major macronutrients in each enterotype. Different letters (A, B) denote statistically significant differences (p-value < 0.05) observed between each enterotype.

We also investigated the proportion of energy intake derived from the three major macronutrients (Fig. 2). Both enterotypes displayed a similar pattern of energy utilization, with carbohydrates constituting the largest source followed by fat and protein. However, individuals with the Sa enterotype exhibited a significantly higher carbohydrate consumption ratio than those with the Ap/Pe enterotype (p < 0.05).

### Correlation between mycobiome enterotype, host dietary habits and metabolic traits

**Figure 3:**
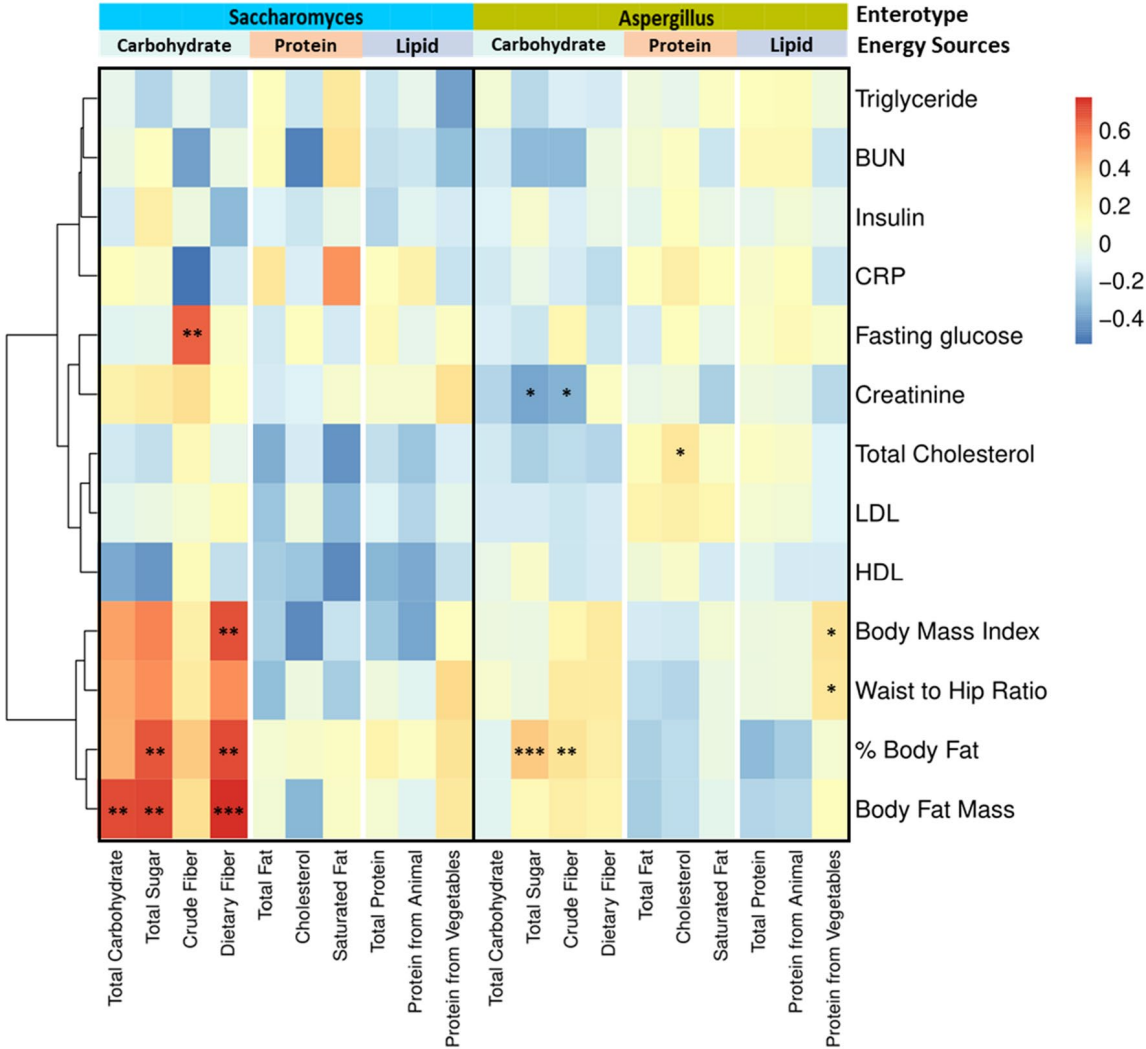
Correlation analysis showing different impacts of dietary intake on host metabolic factors and body composition within distinct enterotype clusters. The Asterisks symbol indicate the false discovery rate (*< 20%, **< 10%, ***< 5%). The color of each box in the matrix signifies the correlation coefficient score (red indicates positive correlation, while blue indicates negative correlation).

Body composition (BMI, WHR, and body fat mass) and clinical metabolic traits are often used as markers in determining host health. Our results showed that the clinical parameters between the Sa and Ap/Pe enterotypes were similar (Table 2). Correlations between dietary intake and several clinical metabolic traits in each enterotype were investigated, with distinct patterns found in the preferred energy sources on each enterotype cluster (Fig. 3). Participants with the Sa enterotype exhibited a strong positive correlation (FDR adjusted, p < 0.05) between total sugar and dietary fiber consumption with body composition parameters including BMI, body fat mass, and percentage of body fat. By contrast, the change of body composition including BMI, Body Fat Mass, Percent Body Fat (PBF%), and hip Ratio (WHR) in volunteers with the Ap/Pe enterotype was primarily driven by total fat intake (correlation coefficient > 0.3, p < 0.05).

**Table 2:** Clinical parameter differences between each enterotype.

Clinical parameter	Enterotype		p-values
	Sa (n = 15)	Ap (n = 45)	
Age	32.33 ± 7.71	29.6 ± 9.37	0.270
Gender (m/f)	(8/7)	(21/24)	0.667
Insulin (µU/mL)	10.53 ± 7.09	8.79 ± 4.95	0.390
Fasting glucose (mg/dL)	85.6 ± 6.01	85.33 ± 7.51	0.890
Total cholesterol (mg/dL)	192.33 ± 31.02	200.78 ± 39.17	0.401
Triglyceride (mg/dL)	91.33 ± 60.9	106.11 ± 66.56	0.434
LDL cholesterol (mg/dL)	126.45 ± 34.75	134.76 ± 38.37	0.442
HDL cholesterol (mg/dL)	53.2 ± 15.8	52.91 ± 11.54	0.949
BUN (mg/dL)	11.8 ± 3.21	12.8 ± 3.33	0.311
Creatinine (mg/dL)	0.81 ± 0.15	0.81 ± 0.19	0.899
CRP (mg/dL)	3.23 ± 4.7	1.83 ± 3.09	0.295
Body Mass Index (BMI)	25 ± 4.61	24.62 ± 4.67	0.787
Body fat mass	21.68 ± 10.19	19.82 ± 9.92	0.544
Percent body fat (PBF%)	29.77 ± 9.3	27.8 ± 10.91	0.503
Waist hip ratio (WHR)	0.88 ± 0.06	0.86 ± 0.06	0.303

Several different behaviors were also observed in host metabolic traits with the intake of specific diets. Among volunteers with the Sa enterotype, fasting glucose, creatinine and total cholesterol, particularly HDL cholesterol in the blood increased with fiber consumption. Conversely, in volunteers with the Ap/Pe enterotype, high levels of creatinine were negatively correlated with the consumption of sugar and crude fiber (FDR adjusted, p < 0.2). Meanwhile, the body fat percent was positively correlated with sugar consumption (FDR adjusted, p < 0.05) while the body mass index and waist to hip ratio was positively correlated with protein consumption (FDR adjusted, p < 0.2). These findings suggested that enterotypes of gut mycobiota dictated the impact of diet on host metabolism.

### Prediction of the mycobiont enterotype functional capabilities related to host metabolism

A predictive analysis of gut mycobiota functional activity was conducted in each enterotype using Phylogenetic Investigation of Communities by Reconstruction of Unobserved States 2 (PICRUST2) software to identify enzymatic activities that might play a role in aiding host diet metabolism. Our findings revealed 868 predicted genes from both groups, with over 70% of these genes associated with metabolism-related processes (Table 3).

**Table 3:** Relative abundance of PICRUSt2 predicted genes in each pathway.

Pathway	Enterotype	
	Sa	Ap
Metabolism	80.51 ± 1.07	79.19 ± 1.25
* Macronutrients*		
Carbohydrate Metabolism	26.96 ± 1.07	26.1 ± 1.4
Protein Metabolism	16.23 ± 0.14	16.25 ± 0.34
Lipid Metabolism	9.37 ± 0.18	9.76 ± 0.36
* Micronutrients*		
Biosynthesis of other secondary metabolites	0.91 ± 0.59	0.83 ± 0.71
Glycan biosynthesis and metabolism	1.56 ± 0.06	1.68 ± 0.12
Metabolism of cofactors and vitamins	8.6 ± 0.15	8.24 ± 0.12
Xenobiotics biodegradation and metabolism	3.7 ± 0.14	3.13 ± 0.2
Genetic information processing	9.02 ± 1.29	10.14 ± 1.29
Environmental information processing	0.11 ± 0.04	0.18 ± 0.06
Cellular processes	1.07 ± 0.04	1.01 ± 0.06
Organismal systems	0.13 ± 0.03	0.16 ± 0.04
Genes and proteins	8.91 ± 0.4	8.7 ± 0.53

**Figure 4:**
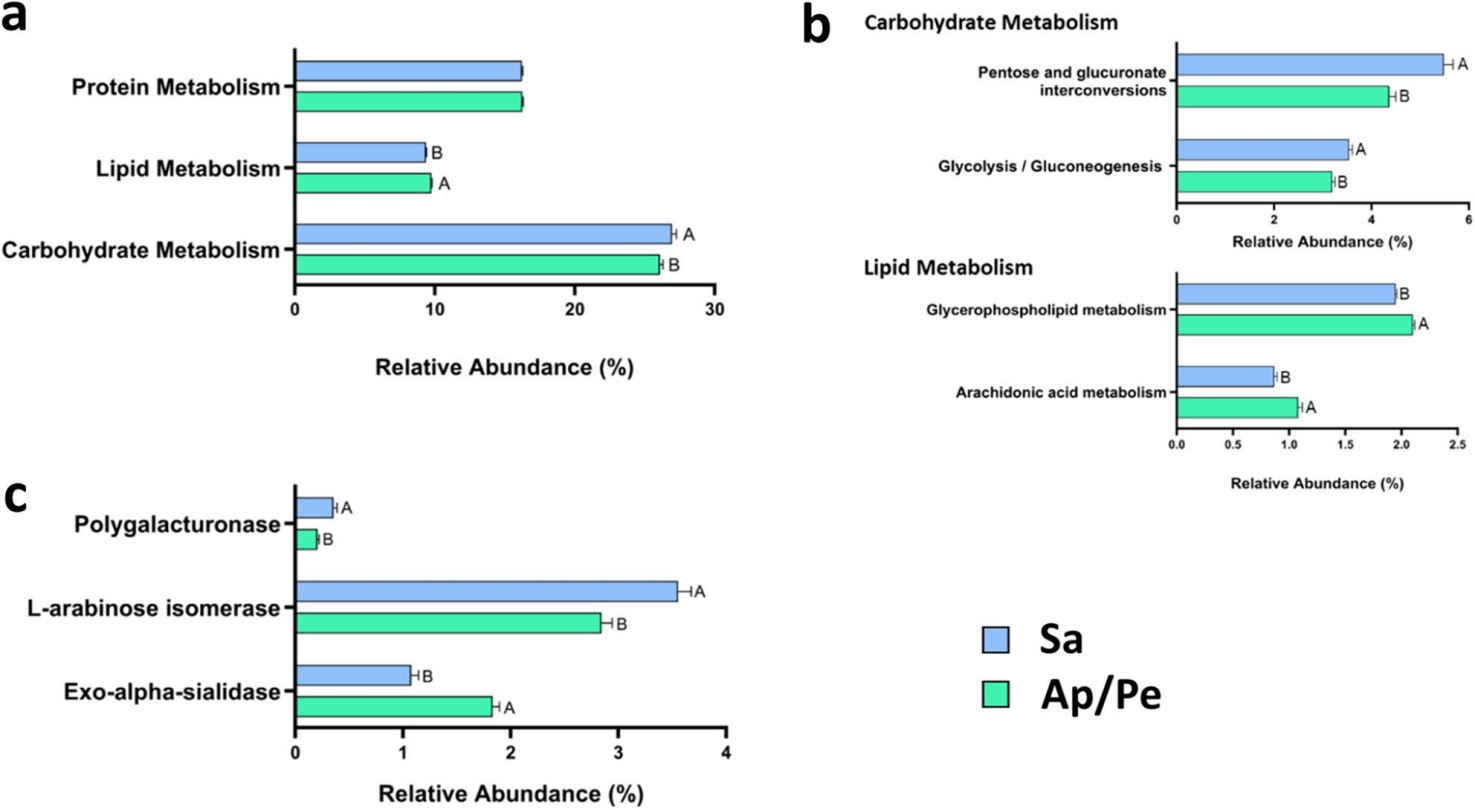
Visualization of PICRUSt 2 predicted relative gene abundances at the metabolism level (**a**), sub-level pathways (**b**), and individual enzymes with highest relative abundances in each sub-level pathway (**c**). Data comprise only statistically significant differences (P-value < 0.05) observed among the enterotypes.

In-depth analyses showed noteworthy differences in relative abundances of genes associated with carbohydrate and lipid metabolism between the two enterotypes (Fig. 4a). Specifically, the Sa enterotype exhibited a significantly higher relative abundance of genes related to carbohydrate metabolism, whereas the Ap/Pe enterotype showed a higher abundance of genes linked to lipid metabolism. However, no significant difference was found in the abundance of genes related to protein metabolism in either group.

A thorough investigation was conducted related to carbohydrate and lipid metabolism to identify the most prominent pathways involved, with pathways exhibiting a relative abundance of at least 3% in carbohydrate metabolism and 1% in lipid metabolism selected for further investigation (Fig. 4b). The fold change of relative abundance in each pathway was also determined between different enterotypes. A comprehensive analysis of the pathways related to carbohydrate and lipid metabolism showed that gene expression involved in the pentose and glucuronate interconversion pathway was significantly higher within the Sa enterotype, while the gene associated with the sphingolipid metabolism pathway exhibited greater prominence within the Ap/Pe enterotype.

When examining individual genes within these pathways (Fig. 4c), results showed that individuals with the Sa enterotype had higher gene expression for l-arabinose isomerase and polygalacturonase. By contrast, individuals with the Ap/Pe enterotype showed a higher abundance of genes encoding exo-alpha-sialidase, an enzyme involved in the cleavage of sialic acid residues. This finding suggested potential implications for host-microbe interactions and nutrient utilization in the gut.

## Discussion

The human gut microbiota comprises a diverse community of microorganisms crucial to overall health and well-being. Previous research explored the correlation between gut bacteria and host metabolism but the role of gut fungi in this process has received less attention. This study investigated the association between gut fungal enterotypes and their influence on metabolism in Thai adults. To the best of our knowledge, no previous study has explored the gut mycobiome (fungal community) profile and its influence on host metabolism in the Thai cohort. Findings suggested that the gut mycobiome might possess important implications in aiding nutrition digestion and serve as a future strategy for weight management and personalized nutrition.

Several studies indicated that different bacteriome enterotypes (i.e., *Prevotella* type and *Bacteroides*-type) are linked to changes in energy metabolism, specifically in energy intake and regulation^8,26,27^. Gut bacteria alterations have been reported to influence host metabolic pathways and were strongly associated with the increased risk of obesity^35–37^. However, limited information exists on how gut fungal enterotypes impact host metabolism.

Our investigations determined that two majors fungal enterotypes dominated the Thai cohort. The Sa enterotype was driven by *Saccharomyces*, while the Ap/Pe enterotype was driven by *Aspergillus* and *Penicillium*. These three fungal genera were identified as major commensals in almost all published studies on gut mycobiome worldwide^2,9,38,39^. Regarding alpha diversity, we observed lower fungi evenness and richness in the Sa enterotype compared to the Ap/Pe enterotype. Higher alpha diversity of fungi has been linked with a greater risk of disease^12^. Our results suggested that this occurrence was more likely due to the blooming of *Saccharomyces* sp., which shifted the gut microbial homeostasis.

The two enterotypes exhibited distinct metabolic behaviors related to nutrient metabolism. Our findings suggested that enterotypes may be responsible for different responses to the same diet. Within the Sa enterotype, a strong positive correlation was found between body composition parameters (such as BMI, WHR, and body fat mass) and fiber consumption. For volunteers with the Sa enterotype, our results also indicated a positive correlation between high fiber consumption and fasting blood glucose levels.

Dietary fiber is recognized for its potential to decrease the risk of obesity by reducing fat absorption and serum cholesterol. However, our study suggests that individuals with the Sa enterotype may possess the ability to extract energy from otherwise indigestible dietary polysaccharides, such as dietary fiber. We speculated that the dietary fiber could also lead to increased caloric release from polysaccharides, resulting in energy deposition in adipocytes^40^. Moreover, a previous in vivo study demonstrated a link between *Saccharomyces* abundance and metabolic disturbances contributing to weight gain in mice ^14^.

Increase in energy intake, together with a less active lifestyle, increases the risk of weight gain and higher fasting glucose levels. Intriguingly, fat consumption was linked to an increase in fasting glucose levels in individuals with the Ap/Pe enterotype but not with the Sa enterotype. High fat and protein intake were found to be the driving forces behind body composition balance for volunteers with the Ap/Pe enterotype. High-fat diets increase the risk of insulin resistance and obesity^41,42^. Thus, a healthier diet was recommended for individuals with this enterotype. *Saccharomyces* has also been found to assimilate lipids^43,44^. This may explain decreases in metabolic activity levels such as insulin and fasting glucose associated with lipid consumption in participants with the Sa enterotype.

Functional analysis indicated that the two enterotypes enriched specific pathways and enzymes differently. The Sa enterotype showed a higher level of the pentose and glucuronate interconversion pathway, particularly specialized in metabolizing less common sugars and fiber components by facilitating their interconversion into forms that can be readily utilized by host metabolism. In this pathway, two enzymes, l-arabinose-isomerase and polygalacturonase, were highly elevated, playing significant roles in efficiently processing l-arabinose and breaking down pectin from plant material.

Evidence also indicated that the pentose and glucuronate interconversion pathway was particularly active during the fed state of metabolism, suggesting that this pathway may be involved in crucial energy storage mechanisms within the host, likely contributing to the formation of glycogen and triglycerides as energy reserves^45^. Our findings aligned with several previous studies which demonstrated that the probiotic strain *Saccharomyces* enhanced the expression of intestinal digestive enzymes, aiding nutrition absorption^46,47^.

The Ap/Pe enterotype exhibited elevated genes related to sphingolipid metabolism, indicating a potential emphasis on the synthesis and degradation of sphingolipids, crucial components of cell membranes and important in cell signaling processes. Other studies also reported that *Aspergillus* and *Penicillium* produced a diverse range of hydrolytic enzymes such as lipase and protease^48,49^. The breakdown of lipid or protein complexes by these enzymes facilitates their enhanced absorption by the host, potentially influencing body composition from fat and protein consumption.

Our findings underscore the potential significance of fungal enterotypes in enabling the host to metabolize various sources of nutrients, thereby potentially impacting overall energy homeostasis. However, this study had limited participants, possibly impacting the results. Moreover, there is a need to prove the capabilities of gut fungi in particular *Saccahromyces* in degrading polysaccharide. Further research involving a larger study population is needed to validate and expand upon our findings.

## Conclusions

This study highlighted the relatively unexplored area of the gut mycobiome and its potential implications on host metabolism within the Thai population. Notably, gut fungal enterotypes significantly influenced nutrient digestion and the overall metabolic activity of the host. Our findings can be used to develop personalized nutrition strategies tailored to each individual’s gut mycobiota enterotype. Given that our study relies on metagenomic analysis and pathway prediction, it's important for future research to confirm the mechanisms we've suggested. In addition, further studies involving larger and more diverse cohorts including those with different lifestyles, are warranted to provide a more comprehensive understanding of the underlying mechanisms at play.

## Methods

### Study design and sample collection

This study formed part of a cohort study conducted to characterize the gut microbiota profiles of the Thai population. A total of 60 healthy study participants aged 20–40 with BMI ranging between 18 and 38 were recruited. All the subjects were free from chronic diseases including cardiovascular disease, diabetes, cancer and inflammatory or digestive disorders. Asthmatics and those consuming more than 21 U/week of alcohol were excluded. The subjects had not taken medications or antibiotics within the preceding three months that might affect lipids, blood clotting or gut microbiota profile. Those who were pregnant and/or breastfeeding were excluded from the study. A previous study^50^ using the same group, analyzed the biochemical blood marker, body composition and nutritional intake behavior of each participant. The nutritional intake and lifestyle behaviors were collected from study subjects using three-day dietary records (3DDR) and semi-quantitative food frequency questionnaires (FFQ). The data were then calculated using INMUCAL-Nutrients version 4 software (Institute of Nutrition, Mahidol University, Thailand).

Fecal samples of all volunteers (approximately 10 g) were collected in a sterilized container, frozen immediately and/or transported with ice packs to the laboratory within 4 h of collection and stored at − 80 °C until further use.

This study was conducted in accordance with the Helsinki Declaration, approved by the Ethics Committee of Kasetsart University (License number 71 COA64/068), and registered with the Thai Clinical Trials Registry (TCTR20220204007). Written informed consent was obtained from all the participants prior to the study.

### Fecal microbial DNA extraction and ITS2 amplicon sequencing

Microbial DNA was extracted using the QIAamp DNA Fast Stool Mini Kit (Qiagen, Hilden, Germany) following protocol Q of the International Human Microbiome Standard (IHMS)^51^. Each fecal sample was homogenized and rinsed twice with phosphate-buffered saline (PBS) pH 8 (1:5 w/v). The fecal pellet was then resuspended in 1.5 mL of ASL lysis buffer (Qiagen, Hilden, Germany) and transferred to a 2 mL screw cap tube containing sterile zirconia beads with diameters of 0.1 mm and 1 mm, weighing 0.3 g for each size (BioSpec, Bartlesville, OK, USA). Physical disruption of microbial cells was performed by running a FastPrep-24 benchtop instrument (MP Biomedicals, Santa Ana, CA, USA) at maximum speed for 8 min and 30 s, with a series of 5 min resting on the ice for every 1 min of beating. The purity and quantity of DNA were determined using a Nanodrop 2000c (Thermo Scientific, Waltham, MA, USA). The genomic DNA was stored at − 20 ℃ until further analysis.

The amplicon sequencing process was carried out by amplifying the ITS2 region using forward primer ITS3 (GCATCGATGAAGAACGCAGC) and reverse primer ITS4 (TCCTCCGCTTATTGATATGC)^52^. The identifier barcode sequence was linked at the 5ʹ end of the forward primer. The amplification process was carried out using an UltraRun LongRange PCR Kit (Qiagen, Hilden, Germany) under the following conditions: Initial denaturation at 95 °C for 2 min; 35 amplification cycles of 95 °C for 30 s, 57 °C for 30 s, 72 °C for 30 s, and a post-extension at 72 °C for 10 min. The PCR product was visualized using 2% agarose, 80 V, 50 min and stained by SafeView™ FireRed (ABM, British Columbia, Canada). Samples with clear and sharp bands were purified using NucleoSpin Gel and PCR Clean-up (Macherey–Nagel, Düren, Germany). The purified samples were then quantified using a Nanodrop 2000c (Thermo Scientific, Waltham, MA, USA) and libraries were prepared by mixing the purified PCR product in equidensity ratios. The mixed library was then sent for sequencing to the outsourcing company (Novogene Co., Ltd., Beijing, China). The sequencing libraries were prepared using NEBNext^®^ Ultra™ DNA Library Prep Kit for Illumina (New England Biolabs, Ipswich, MA, USA) based on the manufacturer’s default protocol. The library quality was assessed using a Qubit 2.0 Fluorometer (Thermo Scientific, Waltham, MA, USA) and Agilent Bioanalyzer 2100 system (Agilent Technologies, Santa Clara, CA, USA). The library was then sequenced on Illumina NovaSeq 6000 (Illumina, San Diego, CA, USA).

### ITS2 sequencing analysis

The ITS2 read pairs were demultiplexed and assigned to each sample based on the unique barcode sequence. All reads were then merged into a single file and the primer sequences were trimmed using the search_pcr2 and fastx_truncate algorithm in USEARCH v11.0.667^53^. Reads shorter than 150 bp or containing expected error more than 1.0 or higher were discarded. The quality-filtered reads were corrected for sequence error by implementing the UNOISE algorithm^54^. An amplicon sequence variants (ASVs) table was produced with USEARCH and then aligned with the demultiplexed reads to recover the abundance. Taxonomy was assigned by the SINTAX algorithm^55^ and the UNITE + INSD dataset for fungi version 8.3 10.05.2021^56^ with an 80% identity cut-off. Microbial diversity metrics were then calculated from the ASVs table with USEARCH. The raw ITS2 amplicon sequences used in this study have been deposited at the NCBI shorts read archive (SRA) with the Bio Project accession number PRJNA1010567.

### Enterotyping the gut mycobiome across study participants

Enterotype clustering was used to group individuals based on their distinct microbial communities. The enterotyping process was performed using hierarchical clustering in PRIMER 7. In brief, the ASV data were normalized by dividing each ASV by the total reads in each sample. The resemblance of gut mycobiota in each sample was then measured by Bray–Curtis similarity. A clustering tree was constructed using a complete linkage algorithm based on the similarity of fungi in each sample.

To determine the robustness of the enterotype clustering obtained from previous step, we then recluster the samples by using the abundance of genus according to the methods suggested by Arumugam et al.^24^. In brief, the optimum cluster number in our study was calculated based on the Calinski-Harabasz (CH) index. The clustering of the first two principal components was then done by using the partitioning around the medoids (PAM) algorithm. All processes were performed in R version 4.3.2 by employing ‘ade4’ and ‘clusterSim’ packages.

### Statistical and bioinformatic analysis

The normality of the dataset was assessed before statistical analysis using the Shapiro–Wilk test in Graph Pad Prism version 9.5.1. Welch’s t-test was utilized for parametric data, with the Kruskal–Wallis or Mann–Whitney test used for non-parametric data. The association between gut mycobiome enterotype clusters and food intake was determined by canonical correlation analysis (CCA) to PCA value from ASV data in PRIMER 7 v.7.0.23, and Spearman correlation analysis was employed to analyze enterotype-specific associations between dietary intake and inflammatory metabolic traits. The correlation analysis was conducted in R version 4.3.2 by employing ‘corrplot’ packages with FDR adjustment for correlation coefficient with significant value p < 0.05.

Determination of the taxa driving each cluster as an enterotype across the sample group was assessed using linear discriminant analysis effect size (LEFSe)^57^. In brief, the unpaired Wilcoxon and two-tailed non-parametric Kruskal–Wallis tests were used to determine any significant differences in the data across the groups. The effect magnitude was then estimated using linear discriminant analysis (LDA) on significantly different data.

The PICRUSt2 pipeline package (v 2.5.1) was used to predict the functional potential of gut microbiomes from the observable ITS sequences^58^. The KEGG pathway levels were inferred by manual curation, based on the EC number of each enzyme lacking compatibility between the KEGG database^59^ and the ITS data. All figures were visualized on ImageGP^60^ and Graph Pad Prism version 9.5.1.

### Supplementary Information

Supplementary Figures.

Supplementary Tables.

## Data Availability

The raw ITS2 amplicon sequences used in this study have been deposited at the NCBI shorts read archive (SRA) with the Bio Project accession number PRJNA1010567.

## References

[1] Zhang F, Aschenbrenner D, Yoo JY, Zuo T (2022). The gut mycobiome in health, disease, and clinical applications in association with the gut bacterial microbiome assembly. Lancet Microbe.

[2] Zhang L, Zhan H, Xu W, Yan S, Ng SC (2021). The role of gut mycobiome in health and diseases. Therap. Adv. Gastroenterol..

[3] Hu J (2022). Gut microbiota signature of obese adults across different classifications. Diabetes Metab. Syndr. Obes..

[4] O'Keefe SJD (2016). Diet, microorganisms and their metabolites, and colon cancer. Nat. Rev. Gastroenterol. Hepatol..

[5] Ost KS, Round JL (2023). Commensal fungi in intestinal health and disease. Nat. Rev. Gastroenterol. Hepatol..

[6] Begum N (2022). Host-mycobiome metabolic interactions in health and disease. Gut Microbes.

[7] Buddhasiri S (2021). Anti-inflammatory effect of probiotic Limosilactobacillus reuteri KUB-AC5 against salmonella infection in a mouse colitis model. Front. Microbiol..

[8] Montenegro J (2023). Exploring the influence of gut microbiome on energy metabolism in humans. Adv. Nutr..

[9] Nash AK (2017). The gut mycobiome of the Human Microbiome Project healthy cohort. Microbiome.

[10] Turunen J, Paalanne N, Reunanen J, Tapiainen T, Tejesvi MV (2023). Development of gut mycobiome in infants and young children: A prospective cohort study. Pediatr. Res..

[11] Jayasudha R (2020). Gut mycobiomes are altered in people with type 2 diabetes mellitus and diabetic retinopathy. PLoS One.

[12] Arunasri K (2020). Mycobiome changes in the vitreous of post fever retinitis patients. PLOS ONE.

[13] Mishima R (2023). Longitudinal gut mycobiota changes in Japanese infants during first three years of life. J. Biosci. Bioeng..

[14] Mims TS (2021). The gut mycobiome of healthy mice is shaped by the environment and correlates with metabolic outcomes in response to diet. Commun. Biol..

[15] Li XV, Leonardi I, Iliev ID (2019). Gut mycobiota in immunity and inflammatory disease. Immunity.

[16] Willis KA (2019). Fungi form interkingdom microbial communities in the primordial human gut that develop with gestational age. Faseb. J..

[17] Flint HJ, Scott KP, Duncan SH, Louis P, Forano E (2012). Microbial degradation of complex carbohydrates in the gut. Gut Microbes.

[18] Skalski JH (2018). Expansion of commensal fungus Wallemia mellicola in the gastrointestinal mycobiota enhances the severity of allergic airway disease in mice. PLoS Pathog..

[19] Briard B, Fontaine T, Kanneganti TD, Gow NAR, Papon N (2021). Fungal cell wall components modulate our immune system. Cell Surf..

[20] Garcia-Rubio R, de Oliveira HC, Rivera J, Trevijano-Contador N (2020). The fungal cell wall: Candida, cryptococcus, and aspergillus species. Front. Microbiol..

[21] Yu D (2022). Dynamics of the gut bacteria and fungi accompanying low-carbohydrate diet-induced weight loss in overweight and obese adults. Front. Nutr..

[22] Forbes JD, Bernstein CN, Tremlett H, Van Domselaar G, Knox NC (2019). A fungal world: Could the gut mycobiome be involved in neurological disease?. Front. Microbiol..

[23] Gu Y (2019). The potential role of gut mycobiome in irritable bowel syndrome. Front. Microbiol..

[24] Arumugam M (2011). Enterotypes of the human gut microbiome. Nature.

[25] Raethong N (2021). Analysis of human gut microbiome: Taxonomy and metabolic functions in Thai adults. Genes.

[26] Cronin P, Joyce SA, O'Toole PW, O'Connor EM (2021). Dietary fibre modulates the gut microbiota. Nutrients..

[27] Jackson MI, Jewell DE (2019). Balance of saccharolysis and proteolysis underpins improvements in stool quality induced by adding a fiber bundle containing bound polyphenols to either hydrolyzed meat or grain-rich foods. Gut Microbes.

[28] Guirro M (2019). Effects from diet-induced gut microbiota dysbiosis and obesity can be ameliorated by fecal microbiota transplantation: A multiomics approach. PLoS One.

[29] Shuai M (2022). Mapping the human gut mycobiome in middle-aged and elderly adults: Multiomics insights and implications for host metabolic health. Gut.

[30] Pérez JC (2021). Fungi of the human gut microbiota: Roles and significance. Int. J. Med. Microbiol..

[31] Mok K (2021). ITS2 sequencing and targeted meta-proteomics of infant gut mycobiome reveal the functional role of Rhodotorula sp. during atopic dermatitis manifestation. J. Fungi.

[32] Limon JJ, Skalski JH, Underhill DM (2017). Commensal fungi in health and disease. Cell Host Microbe.

[33] Hallen-Adams HE, Suhr MJ (2017). Fungi in the healthy human gastrointestinal tract. Virulence.

[34] Kumamoto CA, Gresnigt MS, Hube B (2020). The gut, the bad and the harmless: Candida albicans as a commensal and opportunistic pathogen in the intestine. Curr. Opin. Microbiol..

[35] Boulangé CL, Neves AL, Chilloux J, Nicholson JK, Dumas M-E (2016). Impact of the gut microbiota on inflammation, obesity, and metabolic disease. Genome Med..

[36] Geng J, Ni Q, Sun W, Li L, Feng X (2022). The links between gut microbiota and obesity and obesity related diseases. Biomed. Pharmacother..

[37] Sanmiguel C, Gupta A, Mayer EA (2015). Gut microbiome and obesity: A plausible explanation for obesity. Curr. Obes. Rep..

[38] Raimondi S (2019). Longitudinal survey of fungi in the human gut: ITS profiling, phenotyping, and colonization. Front. Microbiol..

[39] Menglei S (2022). Mapping the human gut mycobiome in middle-aged and elderly adults: Multiomics insights and implications for host metabolic health. Gut.

[40] Kovatcheva-Datchary P (2015). Dietary fiber-induced improvement in glucose metabolism is associated with increased abundance of prevotella. Cell Metab..

[41] von Frankenberg AD (2017). A high-fat, high-saturated fat diet decreases insulin sensitivity without changing intra-abdominal fat in weight-stable overweight and obese adults. Eur. J. Nutr..

[42] Hancock CR (2008). High-fat diets cause insulin resistance despite an increase in muscle mitochondria. Proc. Natl. Acad. Sci..

[43] Dhewantara FX (2016). Cholesterol-lowering effect of beta glucan extracted from saccharomyces cerevisiae in rats. Sci. Pharm..

[44] Girard P, Pansart Y, Verleye M (2014). Anti-hypercholesterolemic effect of Saccharomyces boulardii in the hamster. Pharmacology.

[45] Harding JW, Pyeritz EA, Morris H, White H (1975). Proportional activities of glycerol kinase and glycerol 3-phosphate dehydrogenase in rat hepatomas. Biochem. J..

[46] Moré MI, Vandenplas Y (2018). Saccharomyces boulardii CNCM I-745 improves intestinal enzyme function: A trophic effects review. Clin. Med. Insights Gastroenterol..

[47] Chuang WY, Lin LJ, Hsieh YC, Chang SC, Lee TT (2021). Effects of Saccharomyces cerevisiae and phytase co-fermentation of wheat bran on growth, antioxidation, immunity and intestinal morphology in broilers. Anim. Biosci..

[48] Yang Y (2021). Supplemental aspergillus lipase and protease preparations display powerful bifidogenic effects and modulate the gut microbiota community of rats. Fermentation.

[49] Kumura H (2019). Lipase and protease production of dairy Penicillium sp. on milk-protein-based solid substrates. Int. J. Dairy Technol..

[50] Somnuk S (2023). Metabolic and inflammatory profiles, gut microbiota and lifestyle factors in overweight and normal weight young thai adults. PLoS One.

[51] Costea PI (2017). Towards standards for human fecal sample processing in metagenomic studies. Nat. Biotechnol..

[52] White, T. J., Bruns, T., Lee, S. & Taylor, J. in *PCR Protocols* (eds Michael A. Innis, David H. Gelfand, John J. Sninsky, & Thomas J. White) 315–322 (Academic Press, 1990).

[53] Edgar RC (2010). Search and clustering orders of magnitude faster than BLAST. Bioinformatics.

[54] Edgar RC, Flyvbjerg H (2015). Error filtering, pair assembly and error correction for next-generation sequencing reads. Bioinformatics.

[55] Edgar, R. C. SINTAX: A simple non-Bayesian taxonomy classifier for 16S and ITS sequences. *biorxiv*, 074161 (2016).

[56] Abarenkov K (2021). Full UNITE+INSD dataset for Fungi. UNITE Community..

[57] Segata N (2011). Metagenomic biomarker discovery and explanation. Genome Biol..

[58] Douglas GM (2020). PICRUSt2 for prediction of metagenome functions. Nat. Biotechnol..

[59] Kanehisa M, Goto S (2000). KEGG: Kyoto encyclopedia of genes and genomes. Nucleic Acids Res..

[60] Chen T, Liu Y-X, Huang L (2022). ImageGP: An easy-to-use data visualization web server for scientific researchers. iMeta.

