# Supplementary material for: Preliminary characterization of gut mycobiome enterotypes reveals the correlation trends between host metabolic parameter and diet: a case study in the Thai Cohort
Source: Sci Rep. 2024 Mar 9;14:5805. doi: 10.1038/s41598-024-56585-2 (PMC10924899; doi:10.1038/s41598-024-56585-2)
Supplement: Supplementary file 1 — Supplementary Figures. [file 41598_2024_56585_MOESM1_ESM.docx]

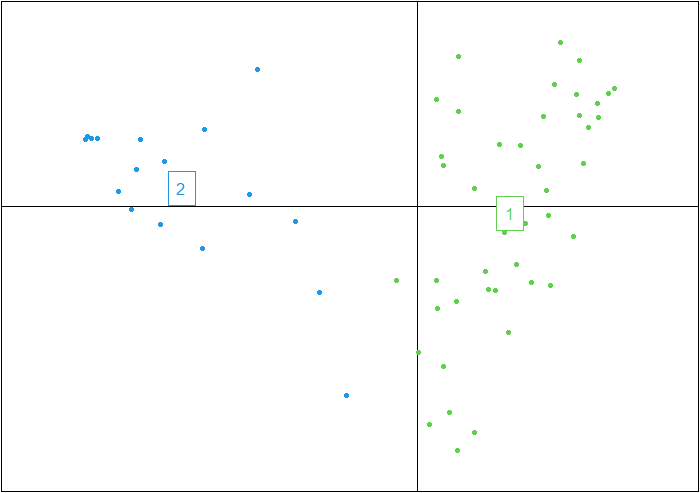
Figure S1. The clusters of enterotype in the studied samples based on the partitioning around medoids (PAM) algorithm. Cluster 1 consisted of 45 samples and cluster 2 consisted of 15 samples.


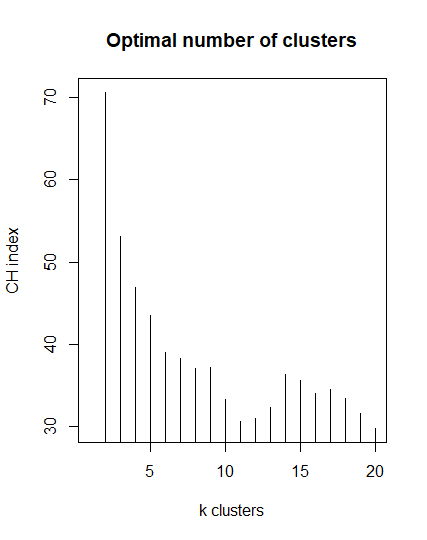
Figure S2. The optimum number of clusters within the studied samples according to the CH index.


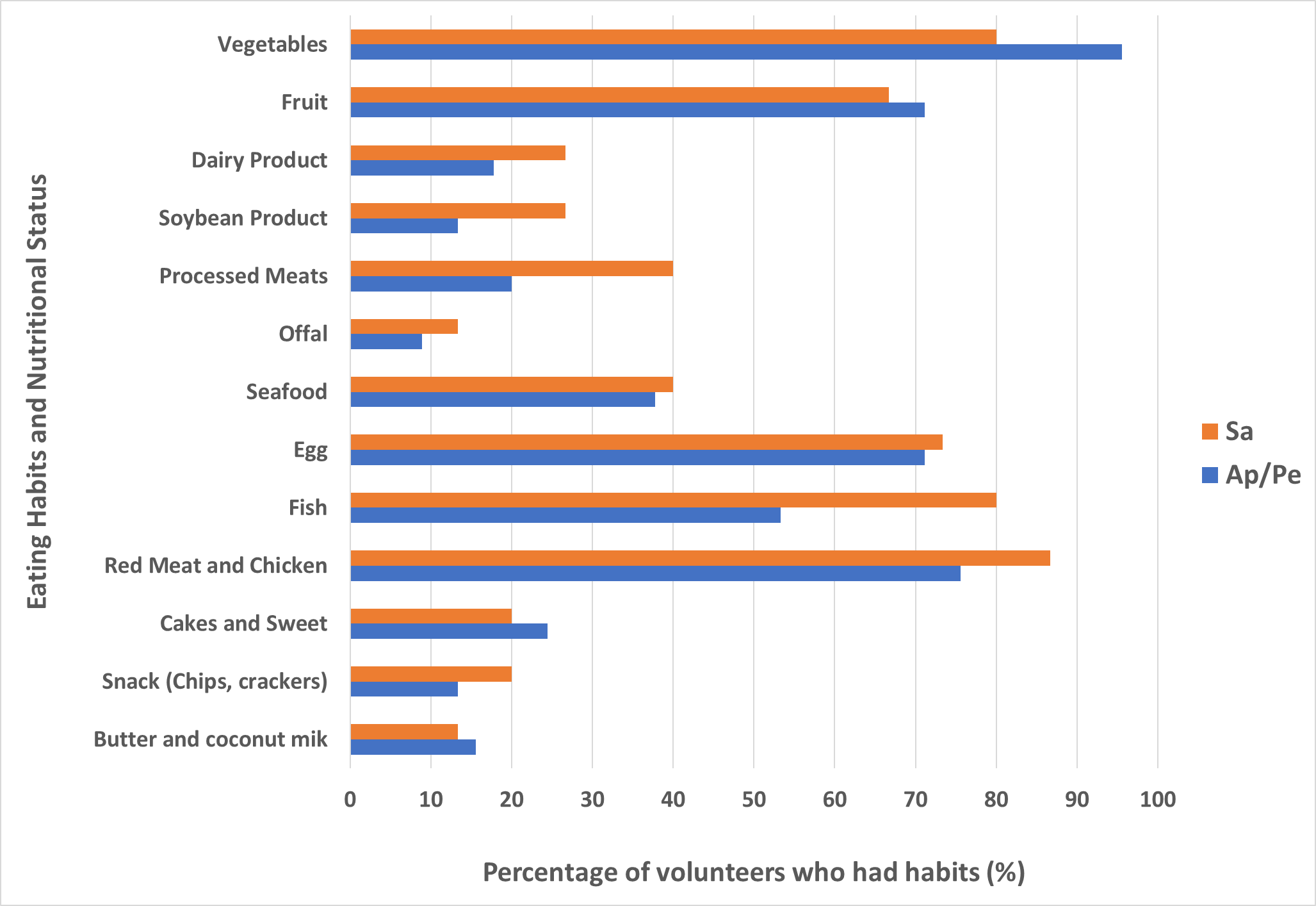

Figure S3. Comparison of eating preferences between individuals in two enterotypes


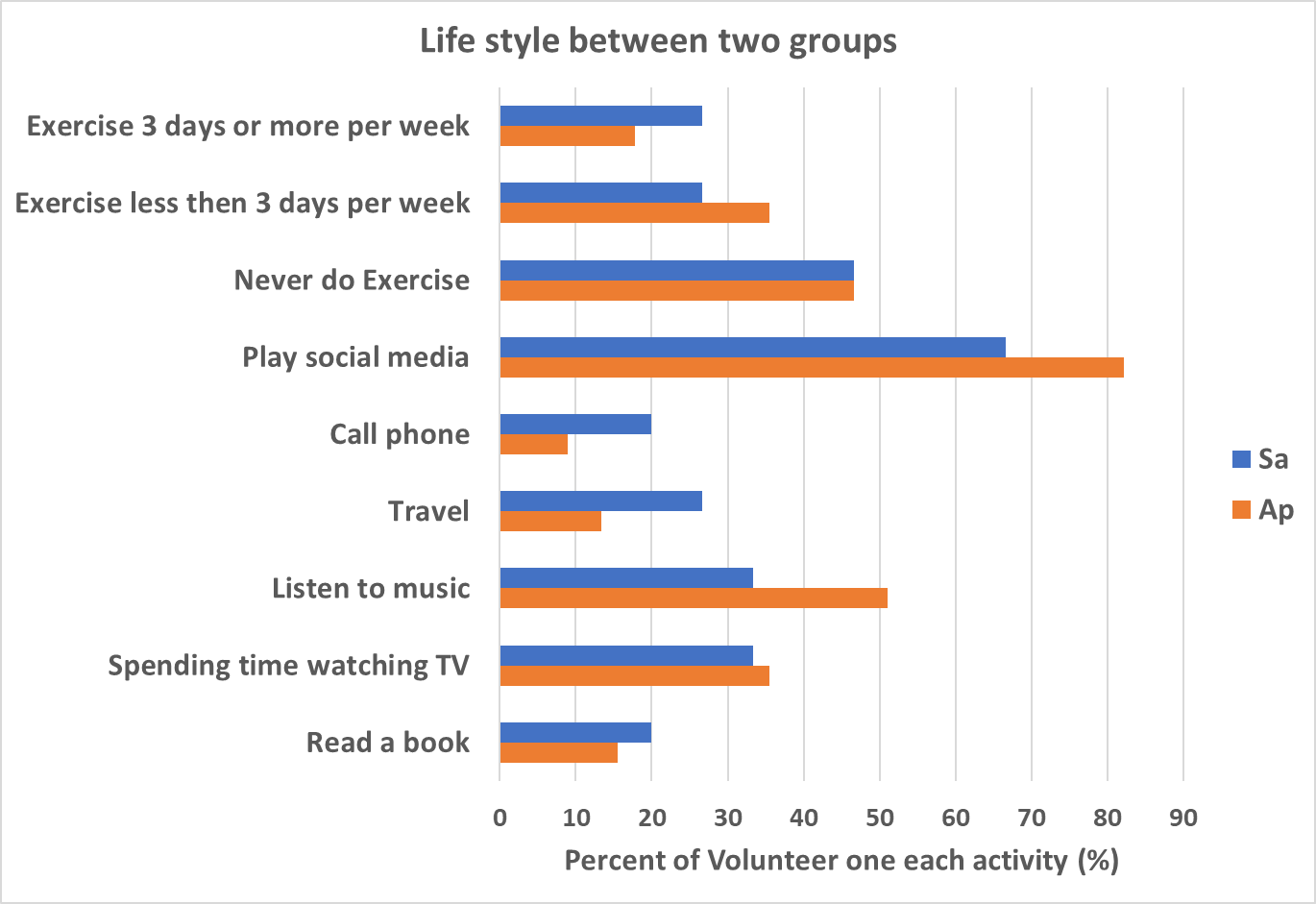


Figure S4. Comparison of activity during daily life between individuals in two enterotypes
