# Supplementary material for: Preliminary characterization of gut mycobiome enterotypes reveals the correlation trends between host metabolic parameter and diet: a case study in the Thai Cohort
Source: Sci Rep. 2024 Mar 9;14:5805. doi: 10.1038/s41598-024-56585-2 (PMC10924899; doi:10.1038/s41598-024-56585-2)
Supplement: Supplementary file 2 — Supplementary Tables. [file 41598_2024_56585_MOESM2_ESM.docx]

| Phylum | Enterotype | | p-value |
| --- | --- | --- | --- |
|  | Sa | Ap/Pe |  |
| Ascomycota | 39.54 ± 49.27 | 79.08 ± 17.32 | 0.191 |
| Basidiomycota | 0.72 ± 0.91 | 18.28 ± 17.64 | **< 0.0001** |
| Chytridiomycota | 0.02 ± 0.08 | 0.02 ± 0.14 | 0.477 |
| Mucoromycota | 0.17 ± 0.61 | 0.26 ± 0.7 | 0.177 |

Supplementary Table 1 List of fungi at phylum level taxa in each enterotype

| Class | Enterotype | | p-value |
| --- | --- | --- | --- |
|  | Sa | Ap/Pe |  |
| Dothideomycetes | 2.34 ± 4.59 | 8.69 ± 15.16 | **0.006** |
| Eurotiomycetes | 6.7 ± 8.4 | 37.57 ± 25.6 | **< 0.0001** |
| Saccharomycetes | 81.54 ± 13.98 | 19.61 ± 20.87 | **< 0.0001** |
| Sordariomycetes | 0.36 ± 0.53 | 1.37 ± 2.06 | **0.001** |
| Agaricomycetes | 0.32 ± 0.46 | 4.25 ± 10.79 | **0.001** |
| Cystobasidiomycetes | 0.43 ± 0.7 | 1.69 ± 3.79 | 0.040 |
| Microbotryomycetes | 0.45 ± 0.93 | 2.01 ± 6.98 | 0.203 |
| Tremellomycetes | 0.51 ± 0.92 | 4.01 ± 8.5 | **0.002** |
| Ustilaginomycetes | 0.23 ± 0.32 | 2.71 ± 4.69 | 0.011 |
| Wallemiomycetes | 1.87 ± 6.12 | 2.5 ± 8.65 | 0.023 |
| Mucoromycetes | 0.17 ± 0.61 | 0.26 ± 0.7 | 0.177 |
| Agaricostilbomycetes | 0.07 ± 0.12 | 0.32 ± 1.16 | 0.495 |
| Malasseziomycetes | 0.02 ± 0.02 | 0.17 ± 0.29 | **0.000** |

Supplementary Table 2 List of fungi at class level taxa in each enterotype

Supplementary Table 3 List of fungi at genera level taxa in each enterotype

| Genera | Enterotype | | p-value |
| --- | --- | --- | --- |
|  | Sa | Ap/Pe |  |
| Saccharomyces | 74.41 ± 18.55 | 4.55 ± 5.81 | **< 0.0001** |
| Aspergillus | 1.89 ± 2.68 | 11.14 ± 10.52 | **< 0.0001** |
| Candida | 5.07 ± 9.57 | 7.86 ± 14.97 | 0.091 |
| Penicillium | 0.57 ± 0.76 | 4.93 ± 4.78 | **< 0.0001** |
| Wallemia | 0.91 ± 2.68 | 2.3 ± 8.32 | **0.008** |
| Hypsizygus | 0.01 ± 0.02 | 3.11 ± 10.93 | 0.012 |
| Rhodotorula | 0.44 ± 0.93 | 1.93 ± 6.98 | 0.179 |
| Trichosporon | 0.13 ± 0.26 | 1.85 ± 6.05 | 0.117 |
| Apiotrichum | 0.03 ± 0.07 | 1.68 ± 6.42 | 0.233 |
| Exophiala | 0.88 ± 1.95 | 0.33 ± 0.55 | 0.352 |
| Pseudozyma | 0.06 ± 0.15 | 1 ± 2.73 | 0.391 |
| Cyrenella | 0.22 ± 0.48 | 0.73 ± 2.29 | 0.745 |
| Cladosporium | 0.1 ± 0.13 | 0.61 ± 1.2 | 0.012 |
| Lodderomyces | 0.45 ± 1.38 | 0.14 ± 0.44 | 0.731 |
| Cystobasidium | 0.06 ± 0.09 | 0.46 ± 0.79 | **0.000** |
| Fusarium | 0.02 ± 0.07 | 0.4 ± 1.84 | 0.208 |
| Sterigmatomyces | 0.05 ± 0.1 | 0.32 ± 1.16 | 0.225 |
| Talaromyces | 0.06 ± 0.07 | 0.28 ± 0.5 | 0.152 |
| Ustilago | 0.03 ± 0.05 | 0.29 ± 1.03 | 0.312 |
| Torulaspora | 0.07 ± 0.19 | 0.23 ± 0.71 | 0.771 |
| Kazachstania | 0.01 ± 0.01 | 0.26 ± 0.94 | 0.053 |
| Volvariella | 0.02 ± 0.02 | 0.24 ± 1.03 | 0.654 |
| Papiliotrema | 0.04 ± 0.13 | 0.19 ± 0.85 | 0.011 |
| Mucor | 0.15 ± 0.59 | 0.07 ± 0.35 | 0.051 |
| Rhizopus | 0.01 ± 0.02 | 0.18 ± 0.62 | 0.295 |
| Pleurotus | 0.03 ± 0.08 | 0.14 ± 0.6 | 0.993 |
| Acremonium | 0 ± 0.01 | 0.16 ± 0.48 | **0.000** |
| Nakaseomyces | 0.02 ± 0.08 | 0.1 ± 0.27 | 0.599 |
| Cutaneotrichosporon | 0.05 ± 0.2 | 0.06 ± 0.18 | 0.073 |
| Cyphellophora | 0.07 ± 0.24 | 0.04 ± 0.24 | 0.391 |
| Bipolaris | 0.01 ± 0.03 | 0.09 ± 0.45 | 0.979 |
| Psathyrella | 0.02 ± 0.07 | 0.08 ± 0.46 | 0.645 |
| Ceriporia | 0 ± 0 | 0.1 ± 0.52 | 0.544 |
| Cyberlindnera | 0.01 ± 0.02 | 0.09 ± 0.34 | 0.057 |
| Spegazzinia | 0 ± 0.01 | 0.08 ± 0.48 | 0.737 |
| Curvularia | 0.01 ± 0.02 | 0.07 ± 0.27 | 0.976 |
| Moniliella | 0.01 ± 0.03 | 0.07 ± 0.3 | 0.260 |
| Naganishia | 0.02 ± 0.07 | 0.05 ± 0.14 | 1.000 |
| Coprinopsis | 0 ± 0 | 0.07 ± 0.35 | 0.685 |
| Kluyveromyces | 0.03 ± 0.09 | 0.04 ± 0.2 | 0.559 |
| Wickerhamomyces | 0.01 ± 0.02 | 0.06 ± 0.23 | 0.050 |
| Phaeosphaeria | 0 ± 0 | 0.06 ± 0.13 | 0.034 |
| Trichoderma | 0 ± 0.01 | 0.05 ± 0.16 | 0.409 |
| Lachancea | 0 ± 0 | 0.05 ± 0.2 | 0.263 |
| Clonostachys | 0 ± 0 | 0.04 ± 0.13 | 0.720 |
| Debaryomyces | 0 ± 0.01 | 0.04 ± 0.09 | 0.074 |
| Emarcea | 0 ± 0 | 0.04 ± 0.24 | 0.866 |
| Lyomyces | 0 ± 0 | 0.04 ± 0.26 | 0.723 |
| Trichomonascus | 0 ± 0 | 0.04 ± 0.24 | 0.857 |
| Auricularia | 0 ± 0.01 | 0.03 ± 0.19 | 0.938 |
| Vishniacozyma | 0 ± 0 | 0.03 ± 0.13 | 0.413 |
| Pichia | 0 ± 0 | 0.03 ± 0.13 | 0.059 |
| Ramichloridium | 0 ± 0 | 0.03 ± 0.1 | 0.386 |
